# Ethylene Oxide: Acute Four-Hour and One-Hour Inhalation Toxicity Testing in Rats

**DOI:** 10.1155/2011/910180

**Published:** 2011-07-13

**Authors:** William M. Snellings, Donald J. Nachreiner, Lynn H. Pottenger

**Affiliations:** ^1^Snellings Toxicology Consulting, LLC, 23 Trails End Lane, Ridgefield, CT 06877, USA; ^2^Inhalation Department, Bushy Run Research Center, R.D. 4, Mellon Road, Export, PA 15632, USA; ^3^Toxicology and Environmental Research and Consulting, The Dow Chemical Company, 1803 Building, Midland, MI 48674, USA

## Abstract

Ethylene oxide was tested on groups of rats for either 4-hour or 1-hour inhalation exposure, followed by 14 days of observation. Groups of five Sprague-Dawley rats/sex were exposed, and clinical signs and mortality were recorded. Clinical signs noted included irregular breathing, absence of certain reflexes, and tremors. Rats that died had moderate to severe pulmonary congestion. The calculated LC_50_ values, reported as ppm by volume (with 95% confidence limits), were as follows. 4-hour LC_50_ values were 1972 (1887 to 2061) ppm for males; 1537 (1391 to 1698) ppm for females; 1741 (1655 to 1831) ppm for the combined sexes. The 1-hour LC_50_ values were 5748 (5276 to 6262) ppm for males; 4439 (4034 to 4884) ppm for females; 5029 (4634 to 5459) ppm for the combined sexes.

## 1. Introduction

Ethylene oxide (EO, CAS no. 75-21-8) is a colorless liquid, boiling at 10.7°C (760 mmHg) and has a characteristic ether-like odor. It is extremely flammable and can form an explosive mixture with air. It is an important industrial chemical intermediate and has limited use as a low-temperature sterilant, mainly for use on medical equipment and spices, because of its very high reactivity characteristic. EO has been classified as a Toxic Inhalation Hazard chemical by the US Department of Transportation [1]. Mortality in acute inhalation studies in laboratory animals has been reported as early as 1930 [2], where irritant effects were also observed in the respiratory passages, nose, and eyes. In addition, ataxia and adverse effects on respiration were noted. Walker and Greeson [3] described an on-purpose exposure with four human volunteers. At approximately 2500 ppm (duration not reported), the odor was not unpleasant, but was slightly irritating to the nasal passages; whereas, exposure at approximately 12,500 ppm was definitely irritating within 10 seconds. 

There are several studies on the 4-hour acute effects from EO exposure to rats published more than 50 years ago. Justification for selection of the Threshold Limit Value, originally set at 100 ppm, from the early years (1946–1956) of ACGIH efforts [4] was based on its irritation potential. In the mid 1950s, Jacobson et al. [5] at the Army Chemical Center reported on the acute effects of EO in exposed male rats. Ten Sprague-Dawley rats were exposed to EO for 4 hours. Signs of irritation, nasal discharge and lacrimation, and adverse effects on respiration were reported. For the majority of the animals, death occurred within 3 days following the single exposure. Histopathology on the lungs revealed congestion, hemorrhage, and edema. The LC_50_ value for the male rat was calculated as 1460 ppm using the Bliss-Finney method. 

Another acute inhalation study was reported by Carpenter et al. [6] on Sherman rats where they reported a 4-hour exposure to 4000 ppm EO. At this significantly higher concentration, mortality of approximately 50% of the tested animals was recorded. A study performed at the same laboratory is mentioned by Sexton and Henson [7], but the only information given is that the 4-hour LC_50_ would be between 8000 ppm (killed 6/6 rats) and 4000 ppm (killed 0/6 rats). In these two early studies, only nominal concentrations (calculated concentrations based on amount evaporated) were determined, which may have contributed to the differences of mortality in the experiments conducted at 4000 ppm. 

It is also noted that RTECS [8] has attributed Deichmann and Gerarde [9] for conducting a 4-hour LC_50_ in rats resulting in an LC_50_ value of 800 ppm. However, this publication by Deichmann has neither a stated study design nor any data to support any LC_50_ values. The following statement is the only information given for LC_50_ values in this publication: “The estimated LC_50_ for 4 hours exposure is approximately 800–1500 ppm (animal studies).” 

Hence, a more robust 4-hour LC_50_ study in rats was conducted because of the discrepancies between the older studies. In addition, since a 1-hour LC_50_ value in rats is needed for certain risk assessments (e.g., DOT, [10]; requirements for shipments and packaging for Toxic Inhalation Hazard materials), a 1-hour LC_50_ study was also conducted. The 1-hour study was conducted using the United States Department of Transportation guideline protocol (1990s) and both studies followed the standards of Federal Insecticide, Fungicide, and Rodenticide Act (FIFRA) Good Laboratory Practice. These studies have been cited [11], and the details of these studies are presented in this current publication.

## 2. Materials and Methods

### 2.1. Materials

#### 2.1.1. 4-Hour

Stainless-steel gas cylinder partially filled with liquid EO (CAS no. 75-21-8) was obtained from Union Carbide Corporation. Results of compositional analysis indicated that the substance was 99.9% pure EO.

#### 2.1.2. 1-Hour

Stainless-steel gas cylinders containing different concentrations of EO in air (ranging from 4000 to 7000 ppm EO) were obtained from Union Carbide Corporation as GC certified levels of EO.

### 2.2. Animals

Young adult male and female Sprague-Dawley albino rats (approximately 6 weeks of age) were obtained from Harlan Sprague-Dawley, Inc., Indianapolis, Ind, USA. Animals were acclimated to facility environment approximately 1 to 2 weeks before treatment and were on a 12-hour light/dark cycle. The approximate group weight range on the first day of exposure for the 4-hour was 220–280 kg for males and 180–195 kg for females; for the 1-hour was 250–285 kg for males and 185–200 kg for females. Food (pelleted Pro Lab RMH #3000, Agway, Inc.) and municipal water were provided *ad libitum *except during exposure. Temperature and humidity were regulated to maintain 18 to 23°C and 40 to 70%, respectively. 

### 2.3. Vapor Generation Methods

#### 2.3.1. 4-Hour

The cylinder of liquid EO was maintained at approximately 35°C in a water bath. The resulting headspace of EO vapor was regulated through stainless-steel tubing and delivered through a calibrated flowmeter into a mixing chamber with filtered-room air before being carried into the inhalation chamber (under negative pressure).

#### 2.3.2. 1-Hour

The EO vapor (certified concentration) was regulated from the cylinder through stainless steel tubing. The vapor was then metered through a calibrated flowmeter into the inhalation chamber, and when necessary, the EO was diluted with filtered-room air before delivery into the inhalation chamber (under positive pressure).

### 2.4. Inhalation Chamber Design and Operation

#### 2.4.1. 4-Hour

The rats were individually housed in wire-mesh cages and exposed (whole body) in a 1300-liter glass and stainless steel chamber (Rochester-type chamber). The chamber shape was rectangular with a pyramidal top and bottom with the EO vapor mixing with filtered air on the inlet side of the chamber. The calibrated chamber airflow rate was approximately 300 L/min.

#### 2.4.2. 1-Hour

The 1-hour exposures were conducted in a 120-liter Plexiglas and stainless steel chamber that was rectangular in shape. Since the exposure chamber was under a positive pressure, this 120-liter chamber was placed in a negative pressure containment system for safety. Because of the short exposure duration, the test vapor atmosphere was generated in the chamber before placement of the animals into the chamber. After maintaining the desired EO target exposure concentration, the animals, 5/sex in a wire-mesh cage (whole body exposure), were quickly inserted into the chamber by a sliding draw mechanism, and at the end of one hour, they were quickly removed. The calibrated chamber airflow rate was approximately 30 L/min.

### 2.5. Inhalation Chamber EO Concentration Analysis and Sampling

#### 2.5.1. 4-Hour and 1-Hour

A Perkin-Elmer Model 8500 or 3920B gas chromatograph (GC) equipped with a flame ionization detector was used to analyze the exposure chamber atmosphere. Calibration of the GC was achieved by injecting gas standards, which were prepared by volumetric dilution of certified EO standards, provided by Union Carbide Corporation. Each exposure chamber atmosphere was analyzed for EO approximately every 15 minutes (1-hour study) or every 30 minutes (4-hour study). Ethylene oxide concentration is reported as ppm by volume (ppm).

### 2.6. Experimental Design

#### 2.6.1. 4-Hour and 1-Hour

No control (air-only) exposures were performed. Body weight and health status were assessed before rats were randomly assigned into groups. Each group contained either 5 male or 5 female rats, unless it was determined that one sex did not have to be exposed to a different concentration based on mortality data from a previous exposure. The chamber temperature and relative humidity were recorded during exposure. Animals were observed for signs of adverse effects during and following the exposure. During the exposure, observations were recorded for all animals as a group, approximately every 10 minutes. During the 14 days following the exposure, all animals were observed at least once daily (typically morning and evening), and any overt clinical signs were recorded. The animals were weighed prior to exposure and on postexposure days 7 and 14. The change in body weight was calculated by subtracting the preexposure value from each successive weight. A complete necropsy was performed on all animals. The survivors were lightly anesthetized with methoxyflurane and killed by exsanguination. The lungs were weighed at necropsy.

### 2.7. Histological Analysis

Lungs were examined histologically from two exposure groups (4-hour study only) in which most of the animals survived (1850 ppm and 1443 ppm, male and female rats, resp.) and from two exposure groups from which most rats died following exposure (2182 ppm and 1850 ppm, male and female rats, resp.). At necropsy, the lungs were fixed by inflating with 10% neutral buffered formalin. The lungs were then embedded in paraffin, sectioned at 5-6 microns, and examined microscopically after hematoxylin and eosin staining.

### 2.8. Statistical Analysis

The mean and standard deviation of the body weights, body weight changes, lung weights (4-hour study only), and exposure concentrations were calculated. No statistical comparisons were made. The LC_50_ values were determined by the moving average method of Thompson [12] for males, females, and the combined sexes. For calculation of the combined sexes, where there was no exposure for a particular sex because it was predetermined from prior exposures that the results would have been either all deaths or no deaths, the appropriate expected ratio (5/5 or 0/5) was used in order to permit calculation of the LC_50_ value for the combined sexes.

## 3. Results and Discussion

### 3.1. Chamber Concentrations

#### 3.1.1. 4-Hour and 1-Hour

A number of atmospheric samples were taken to show that the controls on the vapor generation system were sufficient and resulted in a relatively stable exposure concentration for each exposure group. For the 4-hour study, the mean (±SD) analytical concentrations of EO (*n* = 8) were for the male rats 2182 (±81), 2026 (±50), 1850 (±117) ppm, and for the female rats 1850 (±117), 1637 (±161), and 1443 (±100) ppm. For the 1-hour study, the mean (±SD) analytical concentrations of EO (*n* = 4) were for the male rats 6161 (±37), 5546 (±128), and 4827 (±62) ppm, and for the female rats 4827 (±62), 4202 (±188), and 3966 (±36) ppm.

### 3.2. Animal Observations

#### 3.2.1. 4-Hour

Clinical signs noted included ocular and nasal irritation, irregular breathing, absence of certain reflexes, ataxia, and tremors. No rats died during any of the 4-hour exposure periods. One male rat died within 1 hour following exposure to the highest level (2182 ppm). All remaining deaths occurred within three days following exposure. For all groups, no clinical signs were observed in survivors after postexposure day 5. Tables 1 and 2 summarize clinical signs, mortality rates, and day of death for male and female rats, respectively.

#### 3.2.2. 1-Hour

Clinical signs noted included ocular and nasal irritation, irregular breathing, absence of certain reflexes, ataxia, and tremors. Group observations during exposure for all groups included hyperactivity for approximately the first 10 minutes of exposure followed by hypoactivity. No rats died during the 1-hour exposure period. All remaining deaths occurred within two days following exposure. For all survivors, no clinical signs were observed after postexposure day 2 (males) or day 3 (females). Tables 3 and 4 describe clinical signs, mortality rates, and day of death for male and female rats, respectively.

### 3.3. Body Weights

#### 3.3.1. 4-Hour and 1-Hour

All body weight changes for surviving animals from day 0, the exposure day, showed an increase at postexposure day 7 and day 14. Although no statistics were conducted, the rate of body weight gain was greater in groups that had lower mortality incidence.

### 3.4. Necropsy and Organ Weights

#### 3.4.1. 4-Hour and 1-Hour

No control group was used in this study. The principal gross findings observed in animals that died included diffuse or multifocal discoloration of the lungs and hyperinflation of the lungs. Clear fluid in the thoracic cavity was recorded in one male and one female that died in the highest exposure groups in the 1-hour study. Focal or multifocal color change of the lungs was the only significant gross finding observed in animals euthanized 14 days following the exposure. This finding was observed sporadically. Additional gross findings observed in rats that died or were sacrificed were attributed to autolysis or regarded as incidental findings. Based on laboratory historical findings, all lung weights (determined only in the 4-hour study animals) of surviving rats were in the normal range. Accurate lung weights could not be obtained for animals that died prior to scheduled terminal sacrifice (14 days postexposure). 

### 3.5. Lung Histopathology

#### 3.5.1. 4-Hour (Only)

No control group was used in this study. Based on laboratory historical findings, the lesions noted are attributed to treatment. Lungs from males and females were examined histologically from the highest and the lowest exposure groups, including animals that did not survive until 14 days postexposure. Treatment-related microscopic lesions were seen in all the lungs of males of the 2182 ppm group and all the females of the 1850 ppm group that died, all by day 2 postexposure (Table 5). The lungs from other groups that had mortality were not examined except for one female in the 1443 ppm group (lowest exposure group for this sex) that died. The principal lung lesion observed in all the animals that died (both sexes) was moderate to severe pulmonary congestion. Other lesions which may indicate a toxic effect on the lungs included mild hemorrhage, pulmonary edema, and emphysema, which were noted in some of the animals that died. Pulmonary edema was noted in 2 of the 4 males that died but in none of the females that died. Emphysema was found in one male animal of the 2182 ppm group that died, as well as in the one survivor of this group that was sacrificed 14 days following the exposure. No other treatment-related microscopic lesions were observed in the lungs of this surviving animal nor in the animals of the 1850 ppm (male) and 1443 ppm (female) exposure groups that were sacrificed 14 days following exposure.

### 3.6. LC_50_ Calculations

#### 3.6.1. 4-Hour and 1-Hour

The LC_50_ calculations for both the 4-hour and 1-hour studies are given in Table 6.

## 4. Conclusion

High concentrations of EO do cause death in acutely exposed rats. In the published history of EO use and accidents in the United States, no original citation has been found that reports a human fatality from acute inhalation exposure as would be expected for a toxic inhalation hazardous material. In agreement with this nonevent is a recent review by the National Research Council [11] where it is stated for human toxicity under acute lethality, “No studies were available on lethality attributable to ethylene oxide exposure in humans.” In addition, Thiess [13] summarizes an incident where 22 workers were overcome from a 900-gallon release of EO through a ruptured valve. In an attempt to alleviate the danger as quickly as possible, there was a disregard to donning of any respiratory protection, thus there was direct acute exposure. The durations of these exposures were not reported in detail, but the actions and descriptions during the event indicate significant exposure. These included the following specific examples: attempts by a worker to close the main valve which resulted in his being drenched by a thick jet of liquid EO that coved his head and hands; actions of a foreman when removing several injured workers; actions of a fireman who stood for about 10 minutes near the spray from the rupture. The principle symptoms of acute EO exposure demonstrated were nausea and periodic vomiting (all 22 workers), that commenced a short time after first exposure and persisted for hours and was characterized for some as very severe vomiting. Thiess [13] reports on another 19 illnesses from industrial accidents from 1956 to 1962. He states: “So far according to our knowledge there have been no cases reported of fatalities after inhalation of pure ethylene oxide.”

From the clinical observation and pulmonary histology examination in the rat 4-hour study, it is not known if the resulting mortality was the result of a severe systemic toxicity or due to some mechanism which interferes with normal respiratory function. In both the 1-hour and 4-hour studies, clinical signs of ataxia, tremors, absence of the startle reflex, absence of the tail/toe pinch reflex, and decreased respiration rate were noted, and all of these could have a neurologic effect from EO exposure as a component. In humans, reports of dizziness and repeated vomiting also support a potential neuropharmacologic action of EO [11]. 

Moderate to severe pulmonary congestion was the most significant finding in the animals that died following acute exposure to EO. Caution must be taken in the interpretation of the other lung lesions identified in animals that were found dead. For example, autolysis can mimic pulmonary edema, and diapedesis of red blood cells from congested capillaries can mimic mild hemorrhage, both of which were recorded in this study. Microscopic findings were not significant for the one male rat that survived exposure to the highest exposure level, with the exception of mild multifocal emphysema. Emphysema in this animal may have been due to dyspnea at the time of exposure. In addition, some of these could be agonal changes. These pulmonary findings are similar to those reported by Jacobson et al. [5] for the same 4-hour duration of exposure in rats. 

Past practice has been to conservatively estimate the 1-hour LC_50_, in the absence of animal data, by multiplying a 4-hour LC_50_ value by 2 [10]. The calculated 1-hour LC_50_ value of 2920 ppm for male rats, using this procedure with the previously published 4-hour value of 1460 ppm by Jacobson et al. [5], is considerably lower than the measured value determined in the current study, 5748 ppm. Now with the 1-hour study performed at the same laboratory under similar conditions as the 4-hour study, and where the rats were exposed for the full one hour to the target concentration, a more accurate adjustment factor for ethylene oxide is closer to 3 than 2 for going from the 4-hour to the 1-hour LC_50_. Furthermore, ten Berge et al. [14] observed that following acute inhalation exposure to a range of irritants and systemic toxicants, mortality in several experimental animal species was associated with concentration (*C*) and duration (*t*) by a modification of Haber's rule; *C* ^*n*^ × *t*, where “*n*” is an integer greater than one (interquartile range of mean values, 1.2–2.2). The data presented herein suggest that this is also the case for EO, where *n* is 1.3. 

These two observations are related, since when two exposure paradigms (concentration *C*_1_ and *C*_2_, duration *t*_1_ and *t*_2_) result in the same degree of risk (in this case, LC_50_):

(1) $$C_{1}^{n}\times t_{1}=C_{2}^{n}\times t_{2}.$$

Substituting into (1) for the case, when *t*_2_ = 4 × *t*_1_ and *n* = 1.3:

(2) $$\frac{C_{1}}{C_{2}}=\sqrt[{n}]{\frac{t_{2}}{t_{1}}}=\sqrt[{1.3}]{4}=2.9.$$

Weight of evidence is a key concept in determining reliability of toxicity values, with actual measured data typically attributed extra weight compared with extrapolations. Inclusion of study design details allow for a more thorough evaluation of each dataset, and thus a better determination of reliability for resulting values. Whether additional data support the previous conclusions or not, the increased information contributes to a more reliable overall conclusion. The 1-hour LC_50_ values described herein, determined based on results from 1-hour exposures and using accepted guideline protocols, conducted according to the standards of Good Laboratory Practice, contribute significant weight of evidence to available data for 1-hour acute inhalation toxicity from EO. The 1-hour values reported here of 5748 ppm for male rats, 4439 ppm for female rats, and 5029 ppm for the combined sexes, strengthen the available information overall, and should be considered reliable for any situations requiring acute, 1-hour toxicity values for EO.

## Figures and Tables

**Table 1:** Clinical observations, mortality rates, and day of death for male rats following 4-hour exposure to EO.

Exposure group (ppm)	Clinical signs		Mortality	Number found dead Observation day			
	During exposure or same day following exposure	During five days of postexposure period	Number dead/total	0^a^	1	2	3
2182	Periocular/perinasal/perioral wetness, gasping, audible respiration, hypoactivity	Periocular/perinasal/perioral encrustation, unkempt fur, decreased respiration rate, hypoactivity	4/5	1^b^	2	1	0

2026	Periocular/perinasal/perioral wetness, gasping, audible respiration, hypoactivity, absence tail/toe pinch reflex	Periocular/perinasal/perioral encrustation, unkempt fur, gasping, audible respiration, decreased respiration rate, hypoactivity, tremors,	4/5	0	3	1	0

1850	Periocular/perinasal wetness, gasping, audible respiration, hypoactivity	Perioral/perinasal encrustation, unkempt fur, audible respiration, decreased respiration rate, hypoactivity	0/5	0	0	0	0

^a^Day 0 is exposure day. Days 1, 2, and 3 are days following exposure day.

^b^Number of animals found dead on a particular day.

**Table 2:** Clinical observations, mortality rates, and day of death for female rats following 4-hour exposure to EO.

Exposure group (ppm)	Clinical signs		Mortality	Number found dead Observation day			
	During exposure or same day following exposure	During five days of postexposure period	Number dead/total	0^a^	1	2	3
1850	Periocular/perinasal wetness, gasping, audible respiration, hypoactivity	Periocular/perinasal encrustation, unkempt fur, audible respiration, decreased respiration rate, hypoactivity	5/5	0^b^	3	2	0

1637	Periocular/perinasal/perioral wetness, gasping, audible respiration, hypoactivity, absence tail/toe pinch reflex	Perinasal/perioral encrustation, unkempt fur, audible respiration, decreased respiration rate, hypoactivity, tremors	4/5	0	3	0	1

1443	Periocular/perinasal/perioral wetness, gasping, audible respiration, hypoactivity, tremors	Perinasal encrustation	1/5	0	1	0	0

^a^Day 0 is exposure day. Days 1, 2, and 3 are days following exposure day.

^b^Number of animals found dead on a particular day.

**Table 3:** Clinical observations, mortality rates, and day of death for male rats following 1-hour exposure to EO.

Exposure group (ppm)	Clinical signs		Mortality	Number found dead Observation day		
	During exposure	During two days of postexposure period	Number dead/total	0^a^	1	2
6161 ppm	Absent startle reflex, gasping	Unkempt fur, decreased respiration rate, hypoactivity, ataxia, tremors	4/5	0^b^	3	1

5546 ppm	Absent startle reflex	Unkempt fur, decreased respiration rate, hypoactivity, ataxia, tremors	1/5	0	0	1

4827 ppm	Absent startle reflex	Perioral/perinasal encrustation	0/5	0	0	0

^a^Day 0 is exposure day. Days 1 and 2 are days following exposure day.

^b^Number of animals found dead on a particular day.

**Table 4:** Clinical observations, mortality rates, and day of death for female rats following 1-hour exposure to EO.

Exposure group (ppm)	Clinical signs		Mortality	Number found dead Observation day		
	During exposure	During three days of postexposure period	Number dead/total	0^a^	1	2
4827 ppm	Absent startle reflex	Perinasal wetness, pericular/perioral/perinasal encrustation, unkempt fur, decreased respiration rate, hypoactivity	5/5	0^b^	1	2

4202 ppm		Periocular/perinasal encrustation, unkempt fur, decreased respiration rate, hypoactivity, ataxia, tremors	1/5	0	4	1

3966 ppm		Unkempt fur, decreased respiration rate, hypoactivity, ataxia, tremors	2/5	0	0	1

^a^Day 0 is exposure day. Days 1 and 2 are days following exposure day.

^b^Number of animals found dead on a particular day.

**Table 5:** Lung histopathology of animals that died following 4-hour exposures to EO.

Microscopic diagnosis	Male		Female	
	2182 ppm	1850 ppm	1850 ppm	1443 ppm
Number found dead/sacrificed moribund	4	0	5	1
Lungs				
Congestion, moderate, marked, or severe	4/4	0	5/5	1/1
Pulmonary edema, minimal or moderate	2/4	0	0/5	0/1
Emphysema, moderate	1/4	0	0/5	0/1
Alveolar histiocytosis, mild	1/4	0	0/5	0/1
Hemorrhage, mild	3/4	0	3/5	0/1
Pneumonitis, interstitial, mild	1/4	0	2/5	0/1

**Table 6:** LC_50_ calculations reported in ppm by volume and converted to mg/m^3^.

	Unit of measurement	4-hour LC_50_ (95% confidence limits)	1-hour LC_50_ (95% confidence limits)	Ratio 1-hour/4-hour
Males	ppm	1972 (1887–2061)	5748 (5276–6262)	2.9
	mg/m^3^	3550 (3397–3710)	10346 (9497–11272)	2.9
Females	ppm	1537 (1391–1698)	4439 (4034–4884)	2.9
	mg/m^3^	2767 (2504–3056)	7990 (7261–8791)	2.9
Combined sexes	ppm	1741 (1655–1831)	5029 (4634–5459)	2.9
	mg/m^3^	3134 (2979–3296)	9052 (8341–9826)	2.9
